# Acute pelvic pain following miscarriage heterotopic pregnancy must be excluded: case report

**DOI:** 10.1186/s12873-019-0268-8

**Published:** 2019-10-25

**Authors:** Udit Thakur, Kiran Atmuri, Angelika Borozdina

**Affiliations:** 1 School of Public Health and Community Medicine, University of New South Wales, Kensington, New South Wales Australia; 2 Monash Health, Clayton, Victoria Australia; 3 Royal Women’s Hospital, Parkville, Victoria Australia; 4 Bendigo Health, Bendigo, Victoria Australia

**Keywords:** Heterotopic pregnancy, Miscarriage, Acute pelvic pain, Early pregnancy

## Abstract

**Background:**

Heterotopic pregnancies are increasing in prevalence and this case highlights the importance of excluding the diagnosis in patients with pelvic pain following miscarriage. A known pre-existing intrauterine pregnancy can be falsely reassuring and delay the diagnosis of a potentially life-threatening concurrent ectopic pregnancy.

**Case presentation:**

In this report, we describe a case of spontaneous heterotopic pregnancy in a woman who had initially presented with pelvic pain and vaginal bleeding, and was diagnosed on pelvic ultrasound with a missed miscarriage; a non-viable intrauterine pregnancy. She re-presented 7 days later with worsening pelvic pain and bleeding, and a repeat pelvic ultrasound identified a ruptured tubal ectopic pregnancy in addition to an incomplete miscarriage of the previously identified intrauterine pregnancy. She underwent an emergency laparoscopy where a ruptured tubal ectopic pregnancy was confirmed.

**Conclusion:**

Being a time critical diagnosis with the potential for an adverse outcome, it is important that the emergency physician considers heterotopic pregnancy as a differential diagnosis in patients presenting with pelvic pain following a recent miscarriage. The same principle should apply to pelvic pain in the context of a known viable intrauterine pregnancy or recent termination of pregnancy. A combination of clinical assessment, beta human chorionic gonadotropin levels, point of care ultrasound and formal transvaginal ultrasound must be utilized together in these situations to explicitly exclude heterotopic pregnancy.

## Background

The etiology of pelvic pain following miscarriage is diverse. A heterotopic pregnancy (HP) should be considered amongst the differential diagnoses, which include endometritis, incomplete miscarriage, ruptured ovarian cyst and non-gynecological causes such as appendicitis or urinary tract infection. HP has traditionally been a rare diagnosis in which an intrauterine and ectopic pregnancy occur concurrently. The prevalence of HP is increasing, primarily due to artificial reproductive technology (ART) and pelvic infections [1]. A missed diagnosis of HP can be life-threatening if the ectopic pregnancy ruptures and causes intra-abdominal bleeding.

## Case presentation

**Fig. 1:**
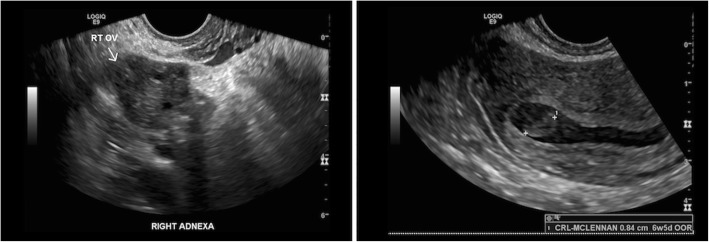
TV US from initial presentation (image on left) showing normal right adnexa. The image on right shows an intra-uterine pregnancy with CRL measurement. The absence of a FHR in a CRL measuring above 7 mm was consistent with a missed miscarriage. The gestational sac measured 20 mm. It was eccentrically placed consistent with a true gestational sac. The gestational sac border was also irregular, a feature of a non-viable pregnancy

A 33-year-old female, gravidity two and parity one, presented to the Emergency Department with acute-onset sharp pelvic pain, right worse than left, and mild vaginal bleeding. She was found to be pregnant with a beta human chorionic gonadotropin (β-hCG) level of 1442 mIU/ml. The pregnancy was spontaneous and unplanned; she was breastfeeding 6 months after a normal vaginal delivery and using the oral contraceptive pill, levonorgestrel, 30mcg daily. She had no significant past gynecological, medical or surgical history. She underwent a formal departmental transvaginal ultrasound (TV US) verified by a Consultant Radiologist that identified an intrauterine pregnancy (IUP) with crown rump length of 8.5 mm without cardiac activity, consistent with 6 + 5 weeks gestation. This met the widely accepted ultrasound criteria for a missed miscarriage [2]. There was a physiological corpus luteal cyst in the left ovary, and the right ovary was normal. After observation overnight, she was discharged for expectant management of the miscarriage as she was clinically stable and had a falling β-hCG from 1442 mIU/ml to 915 mIU/ml over 24 h. She re-presented to the Emergency Department 7 days later with worsening pelvic pain, mostly right sided, and ongoing mild vaginal bleeding. She was hemodynamically stable and mildly tender to palpate in the left iliac fossa. The β-hCG had risen to 2267 mIU/ml. Her hemoglobin dropped to 12.4 g/dL, previously 15.0 g/dL. The Emergency Physician requested a formal departmental TV US, which revealed a new solid right adnexal mass measuring 64x60x40mm with internal vascularity and adherent to the right ovary (Fig. 1). There was a large volume of fluid in the pelvis with low-level echoes. The left-sided corpus luteal cyst was again visualized. An endometrial cavity lesion of 16x9x16mm was thought to be retained products of conception. A diagnosis was made of a HP, with a suspected ruptured tubal ectopic pregnancy and retained products of conception from an incomplete miscarriage of the previously identified non-viable intrauterine pregnancy. A gynecology consult was summoned and the patient was transferred to theatre for surgery.

**Fig. 2:**
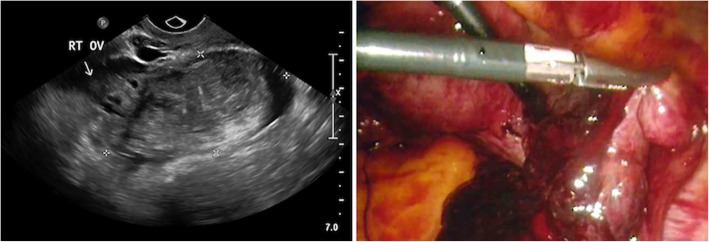
TV US (image on left) shows a 64 × 60 × 40 mm solid appearing mass in the right adnexa separate to the right ovary and free pelvic fluid. At laparoscopy, there was a ruptured right fallopian tube infundibular ectopic pregnancy with haemoperitoneum

A diagnostic laparoscopy was performed with dilation and curettage. A 100 ml hemoperitoneum and ruptured right fallopian tube infundibular ectopic pregnancy adherent to the right ovary was identified (Fig. 2). A right salpingectomy was performed. Histology confirmed a ruptured tubal ectopic pregnancy and endometrial curettings confirmed retained products of conception. She had an uncomplicated recovery. Her follow-up β-hCG level was negative.

## Discussion

HP has traditionally been a rare diagnosis where there is a concurrent intrauterine and ectopic pregnancy. It can be potentially life-threatening if the ectopic ruptures and causes intra-abdominal bleeding. The case highlights the importance of explicitly excluding a HP in all patients presenting with pelvic pain after miscarriage. Such vigilance must also apply to patients presenting with a known viable IUP, and also following a termination of pregnancy.

HP’s are estimated to occur in 1 in 30,000 pregnancies in spontaneous conceptions and increases to 1 in 100 pregnancies if associated with ART, tubal ligation, pelvic inflammatory disease or non-infectious causes of tubal damage such as endometriosis [1]. The patient discussed was using a low-dose progesterone-only pill, which when contraception fails and ovulation occurs may be associated with ectopic pregnancy due to impaired fallopian tube motility [3, 4]. The patient otherwise had no known predisposing factors for a HP.

A delay in diagnosis is more likely to require operative intervention such as salpingectomy [5]. A review of delayed or misdiagnosed HP and ectopic pregnancies identified factors contributing to delayed diagnosis could be classified under 3 categories: (1) clinician factors: unawareness of HP as a diagnostic entity due to its rarity in spontaneous pregnancies, false re-assurance from the presence of a confirmed IUP and patients missed to follow-up due to misinterpretation of β-hCG trends; (2) patient factors: non-compliance with follow-up, and (3) complicated presentations having atypical symptoms, such as GI symptoms [6].

The most common presenting symptom of a HP is unilateral pelvic pain, and others include vaginal bleeding and presyncope [3]. Patients may also report persisting pregnancy symptoms after a miscarriage or termination of pregnancy. Up to 13% of patients may present with signs of shock [5]. Clinical signs include vaginal bleeding, abdominal tenderness to palpation, peritoneal irritation, an enlarged uterus greater than 8 week size, cervical motion tenderness and tender adnexal mass on vaginal examination [7]. These non-specific symptoms and signs may be erroneously attributed to a co-existing IUP, and exemplify the difficulty in clinically diagnosing HPs. It is important to obtain a gynaecology opinion for any patients who re-present with undifferentiated pain [7].

β-hCG levels and TV US together play a critical role in excluding a HP. After a miscarriage, a positive β-hCG result may be normal in the resolution of the pregnancy. Whilst β-hCG levels can be assessed 3 weeks after miscarriage to ensure no molar pregnancy, there is no recommendation to routinely assess β-hCG levels in the follow-up of a miscarriage to exclude heterotopic pregnancy [2]. If a β-hCG level is performed, an abnormal rise or fall in the quantitative β-hCG level may be suggestive – not diagnostic – of a HP. β-hCG levels are expected to fall following a miscarriage or termination of pregnancy. Higher initial β-HCG levels are associated with a more rapid decline, however, in general, by 48 h β-HCG levels should fall by 35% and by the seventh day it is expected that β-hCG levels fall between 60 and 84% [8]. The rate of β-hCG decline is slower in ectopic pregnancies. In the case presented, the β-hCG level after the miscarriage had risen leading to suspicion of a HP. Interestingly, one in three ectopic pregnancies have occurred in the presence of appropriately falling or rising β-hCG levels [9], highlighting the importance of not depending on trends in β-hCG levels in isolation to exclude a HP.

Formal departmental TV US underpins the exclusion of a HP. However, it is essential to note that Emergency Physician (EPs) performed point-of-care ultrasound (PPOCUS) may play a critical role in risk stratification. PPOCUS has a greater than 99% sensitivity and specificity in excluding ectopic pregnancies, but this is through the visualisation of an IUP on transabdominal imaging, which may provide false reassurance and delay the diagnosis of a concurrent extrauterine pregnancy [10]. PPOCUS may play a more useful role in determining patients needing prompt operative care – in those with ruptured ectopic pregnancy. Rather than focusing on identifying an IUP, visualisation of fluid in the hepatorenal space (Morison’s pouch) by PPOCUS has been found to be strongly predictive of the need for operative intervention with a positive likelihood ratio of 112 [11]. In an appropriately trained EP, PPOCUS takes less than 5 min to complete [11]. A recent meta-analysis found that there are significant time savings with PPOCUS, especially at night where access to formal departmental US is limited [12]. If PPOCUS does not identify free fluid, a formal departmental TV US to assess the adnexa should then be arranged, with the urgency dependant on clinical concern and resources. Identifying of an IUP is re-assuring but a systematic assessment of the adnexa must occur to exclude an ectopic pregnancy, and suspicious adnexal masses should undergo further follow-up for change in appearance or size. In the case discussed, there was only an interval of 7 days between presentations and imaging of the right adnexa which highlights the speed at which ectopic pregnancies may develop and rupture. Tubal ectopic pregnancies make up 95% of ectopic pregnancies [3]. Findings of a tubal ectopic on TV US include an empty uterus with an endometrium less than 8-10 mm, free fluid in the pelvis, pseudo-gestational sac in up to 20% of cases, and a complex adnexal mass of varying appearance (inhomogeneous mass, hyperechoic ring, distinct gestational sac with or without a yolk sac and fetal heart) moving separate to the ovary [13].

## Conclusion

Delayed diagnosis of a HP is pertinent for the Emergency Physician as the consequences to the patient can be dire. This case highlights the importance of maintaining a high index of suspicion for a HP in patients with pelvic pain after miscarriage, with the same principle applied to those with a recent termination of pregnancy or known IUP. A combination of history, examination, β-hCG levels, PPOCUS and formal TV US should be considered to explicitly exclude HP in these patients.

## Data Availability

Data sharing is not applicable to this article as no datasets were generated during the study.

## Abbreviations

ART: Artificial reproductive technology
EP: Emergency physician
HP: Heterotopic pregnancy
PPOCUS: Physician performed point-of-care ultrasound
TV US: Transvaginal ultrasound
β-hCG: beta human chorionic gonadotropin
